# Pre-treatment lysis time of plasma-derived fibrin clots and bleeding in patients on oral anticoagulants for atrial fibrillation in the ARISTOTLE trial

**DOI:** 10.1093/eurheartj/ehaf347

**Published:** 2025-05-30

**Authors:** William A E Parker, Thomas A Nelson, Justin Lee, Heather M Judge, Ramzi A Ajjan, Johan Westerbergh, Agneta Siegbahn, Christina Christersson, John H Alexander, Renato D Lopes, Christopher B Granger, Elaine M Hylek, Lars Wallentin, Robert F Storey

**Affiliations:** Cardiovascular Research Unit, Division of Clinical Medicine, University of Sheffield, Beech Hill Road, Sheffield S10 2RX, UK; NIHR Sheffield Biomedical Research Centre, Sheffield Teaching Hospitals NHS Foundation Trust, Glossop Road, Sheffield S10 2JF, UK; South Yorkshire Cardiothoracic Centre, Sheffield Teaching Hospitals NHS Foundation Trust, Herries Road, Sheffield S5 7AU, UK; Cardiovascular Research Unit, Division of Clinical Medicine, University of Sheffield, Beech Hill Road, Sheffield S10 2RX, UK; NIHR Sheffield Biomedical Research Centre, Sheffield Teaching Hospitals NHS Foundation Trust, Glossop Road, Sheffield S10 2JF, UK; South Yorkshire Cardiothoracic Centre, Sheffield Teaching Hospitals NHS Foundation Trust, Herries Road, Sheffield S5 7AU, UK; NIHR Sheffield Biomedical Research Centre, Sheffield Teaching Hospitals NHS Foundation Trust, Glossop Road, Sheffield S10 2JF, UK; South Yorkshire Cardiothoracic Centre, Sheffield Teaching Hospitals NHS Foundation Trust, Herries Road, Sheffield S5 7AU, UK; Cardiovascular Research Unit, Division of Clinical Medicine, University of Sheffield, Beech Hill Road, Sheffield S10 2RX, UK; NIHR Sheffield Biomedical Research Centre, Sheffield Teaching Hospitals NHS Foundation Trust, Glossop Road, Sheffield S10 2JF, UK; LIGHT laboratories, Leeds Institute of Cardiovascular and Metabolic Medicine, University of Leeds, Clarendon Way, Leeds LS2 3AA, UK; Uppsala Clinical Research Center, Uppsala University, Dag Hammarskjölds Väg, 751 85 Uppsala, Sweden; Department of Medical Sciences, Uppsala University, 751 85 Uppsala, Sweden; Department of Medical Sciences, Uppsala University, 751 85 Uppsala, Sweden; Division of Cardiology, Duke Clinical Research Institute, Duke University, W Morgan St, Durham, NC 27701, USA; Division of Cardiology, Duke Clinical Research Institute, Duke University, W Morgan St, Durham, NC 27701, USA; Division of Cardiology, Duke Clinical Research Institute, Duke University, W Morgan St, Durham, NC 27701, USA; Department of Medicine, Boston University School of Medicine, East Concord St, Boston, MA 02118, USA; Uppsala Clinical Research Center, Uppsala University, Dag Hammarskjölds Väg, 751 85 Uppsala, Sweden; Department of Medical Sciences, Uppsala University, 751 85 Uppsala, Sweden; Cardiovascular Research Unit, Division of Clinical Medicine, University of Sheffield, Beech Hill Road, Sheffield S10 2RX, UK; NIHR Sheffield Biomedical Research Centre, Sheffield Teaching Hospitals NHS Foundation Trust, Glossop Road, Sheffield S10 2JF, UK; South Yorkshire Cardiothoracic Centre, Sheffield Teaching Hospitals NHS Foundation Trust, Herries Road, Sheffield S5 7AU, UK

**Keywords:** Atrial fibrillation, Anticoagulation, Fibrinolysis, Bleeding

## Abstract

**Background and Aims:**

Oral anticoagulation reduces stroke risk in patients with atrial fibrillation (AF) but increases bleeding. Longer fibrin clot lysis time has been shown to predict adverse cardiovascular outcomes in acute coronary syndromes. This study explored relationships between fibrin clot lysis time at randomization and clinical outcomes in patients with AF enrolled in the Apixaban for Reduction in Stroke and Other Thromboembolic Events in AF (ARISTOTLE) trial.

**Methods:**

Plasma samples were obtained from anticoagulation-naïve participants, before initiation of study medication (*n* = 1841). Fibrin clot turbidimetry was performed, and lysis time determined. Associations between lysis time and characteristics, biomarkers, and on-treatment bleeding and cardiovascular events were assessed by lysis time quartile (Q1-4, shortest to longest).

**Results:**

A shorter lysis time was associated with being older, male, permanent AF, lower body mass index, estimated glomerular filtration rate and C-reactive protein, and higher N-terminal pro-B-type natriuretic peptide. Major and clinically relevant non-major bleeding was significantly more frequent in lysis time Q1 vs. Q4 [6.3%/yr vs. 2.1%/yr; HR, 2.99 (95% CI, 1.75–5.12); *P* = .001], including after multifactorial adjustment [HR, 2.61 (1.45–4.69); *P* = .016]. Those in Q2 and Q3 had intermediate bleeding risk vs. Q4 [HR, 2.21 (1.27–3.87); 2.08 (1.18–3.66) respectively], suggesting a graduated effect. Treatment allocation to apixaban vs. warfarin did not affect the relationship between lysis time and bleeding (interaction-*P* = .80). There was no significant association between lysis time and a composite of cardiovascular death, stroke, systemic embolism or myocardial infarction.

**Conclusions:**

Shorter pre-treatment fibrin clot lysis time independently predicted higher bleeding risk in patients receiving oral anticoagulation for AF.

## Structured Graphical Abstract

**Structured Graphical Abstract:**
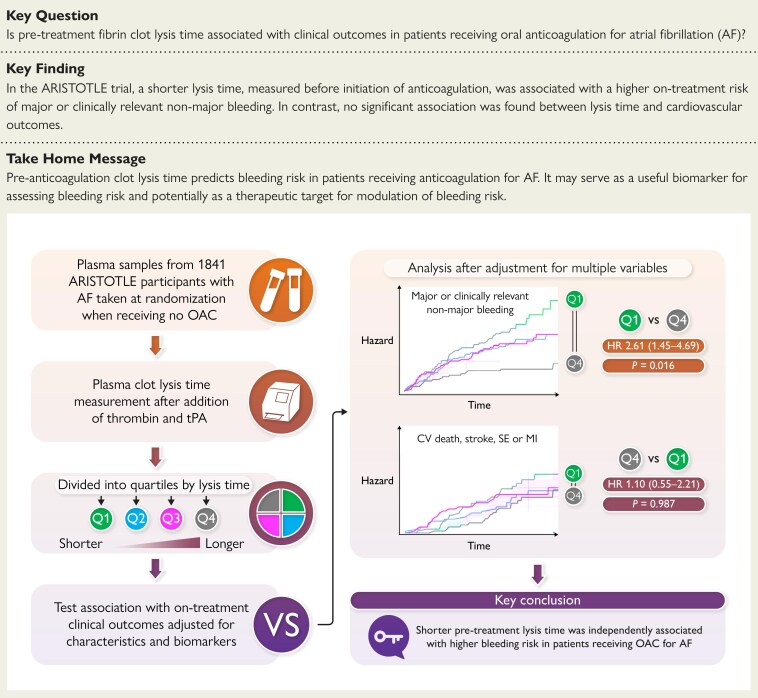

**See the editorial comment for this article ‘Fibrin clot lysis predicts anticoagulant-associated bleeding in atrial fibrillation: ready for clinical use?’, by H. ten Cate and J. ten Berg, https://doi.org/10.1093/eurheartj/ehaf458.**

## Introduction

Atrial fibrillation (AF) is the most common sustained cardiac arrhythmia and a major risk factor for cardioembolic stroke or other systemic embolism (SE).^1,2^ This thromboembolic risk is likely related not only to stasis of blood in the left atrium, and left atrial appendage in particular, but also atrial myopathy and a hypercoagulable local state that may precede the development of arrhythmia.^3^

Oral anticoagulation (OAC) reduces the risk of cardioembolic stroke but increases the risk of bleeding, including major bleeding.^4,5^ Risk scores are available to weigh up these risks, the net clinical effect favouring OAC except in those with the lowest ischaemic risk.^6^ In patients with AF, use of a direct-acting OAC (DOAC), such as the factor Xa inhibitor apixaban, is recommended as first-line over a vitamin K antagonist (VKA) such as warfarin.^1^ In the Apixaban for Reduction in Stroke and Other Thromboembolic Events in Atrial Fibrillation (ARISTOTLE) trial, the rate of the primary outcome of stroke or SE was significantly lower in the group randomized to receive apixaban compared with warfarin.^7^ Apixaban was also associated with a 31% lower rate of International Society on Thrombosis and Haemostasis (ISTH) major bleeding and an 11% lower rate of all-cause mortality.

Coagulation is a dynamic, multifaceted process that includes simultaneous clot formation and lysis, in order to limit extension of the blood clot and resulting in a net effect of varying degrees of fibrin deposition. These processes can be studied using acellular fibrin clot turbidimetry.^8^ Fibrin clot dynamics have previously been associated with clinical outcomes, particularly in the context of acute coronary syndromes (ACS).^9,10^ Notably, in a substudy of the PLATelet inhibition and patient Outcomes (PLATO) trial, which randomized aspirin-treated patients with ACS to receive the platelet P2Y_12_ inhibitors ticagrelor or clopidogrel, a longer time from peak turbidity to 50% lysis (lysis time) was independently associated with a higher risk of the trial composite primary endpoint of cardiovascular death, myocardial infarction (MI), or stroke.^11^ There was no significant association between lysis time and bleeding outcomes in that cohort.

Whilst previous analyses of samples taken during the ARISTOTLE trial have studied coagulation markers in those receiving warfarin and apixaban,^12^ the impact of lysis time on clinical outcomes in patients with AF has not been determined. We therefore explored the association between baseline lysis time, before starting anticoagulation, and on-treatment clinical outcomes in patients with AF participating in the ARISTOTLE trial.

## Methods

### Study design

The design of the ARISTOTLE trial has been reported in detail previously, including particulars of ethical review and regulatory compliance.^7,13^ Briefly, 18 201 patients with AF and at least one additional risk factor for stroke were randomized to receive apixaban 5 mg twice daily [or 2.5 mg if meeting ≥2 of: age ≥80 years, body weight <60 kg, or a serum creatinine level of 1.5 mg/dL (133 μmol/L)] or warfarin (target international normalized ratio 2.0–3.0) in a double-blind trial with double-dummy placebo control. The primary efficacy outcome was ischaemic or haemorrhagic stroke or SE. The primary safety outcome was ISTH major bleeding, defined as clinically overt bleeding accompanied by a decrease in the haemoglobin level of at least 2 g/dL or transfusion of at least two units of packed red cells, occurring at a critical site, or resulting in death. The rate of a composite of major and clinically relevant non-major bleeding was also adjudicated and reported. Clinically relevant non-major bleeding was defined as clinically overt bleeding that did not satisfy the criteria for major bleeding and led to hospital admission, medical or surgical treatment, or a change in antithrombotic therapy.^14^

### Biomarker substudy

**Figure 1:**
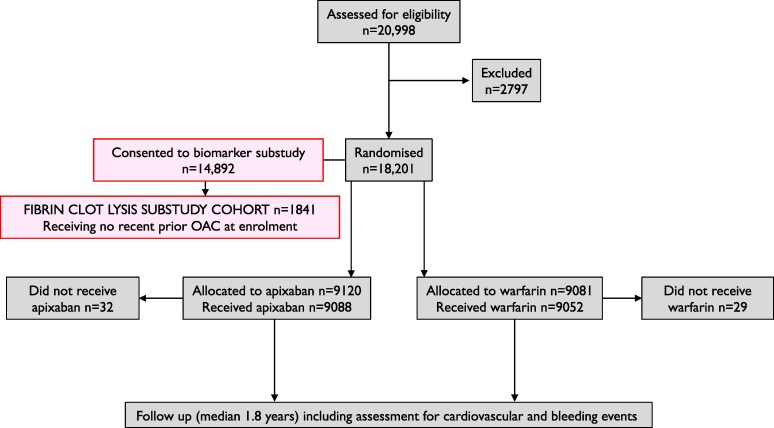
Overall design of the ARISTOTLE fibrin clot lysis substudy in the context of the main trial. OAC, oral anticoagulant

Of the 18 201 participants in ARISTOTLE, 14 892 additionally gave written informed consent to participate in a biomarker substudy that included blood samples taken into citrate tubes on ice at the time of randomization. Plasma was produced by centrifugation and stored at −20°C at participating centres before shipping to Uppsala Clinical Research Centre (UCR) for aliquoting and storage at −70°C. Prior to randomization, 57% were receiving or had previously received a VKA, the remainder not having been exposed to an anticoagulant. For the present study, only samples from participants without prior VKA exposure were analysed in order to avoid potential confounding from the effects of VKA on fibrin clot lysis (*Figure 1*). Sufficient plasma samples were available for 1841 participants. Prior to the fibrin clot analyses, all samples were shipped to the Sheffield Cardiovascular Research Unit in a single batch and stored at −80°C. There were a limited number of plasma samples from patients who had a stroke/SE event due to a previous case-control study.^15^ Numbers of events included in the analysed cohort are provided in Supplementary data online, *Table S1*. The median (IQR) follow-up of participants included in the fibrin clot substudy was 2.0 (1.8–2.3) years.

### Fibrin clot turbidimetry

Turbidimetric clot formation and lysis assays were performed in 96-well plates in duplicates as described previously.^11^ Plasma samples were removed from storage at −80°C and thawed in a water bath at 37°C for 10 min, before inverting several times to ensure mixing. Samples were then aliquoted in duplicate into a flat-bottomed polystyrene 96-well plate (25 μL in each well). Pooled plasma for quality control was created by mixing equal volumes of plasma from 11 healthy volunteers who had given their written consent for venepuncture and blood sample processing for the purposes of research (University of Sheffield Research Ethics Committee, reference 031330). Quality control plasma was used on each plate.

A tris-containing buffer solution (pH, 7.4; NaCl, 100 mmol/L; Tris, 50 mmol/L) was used for dilution of 1 mol/L-calcium chloride (CaCl_2_), tissue plasminogen activator (tPA) and thrombin. Separate lysis and activation mixes were added sequentially to the plasma samples in the 96-well plate: 75 μL lysis mix containing tPA (final concentration, 83.5 ng/mL) was added prior to 50 μL activation mix (thrombin 0.03 IU/mL and CaCl_2_ 7.5 mmol/L; final concentrations).

High-throughput turbidimetric analysis was performed using a plate reader measuring light absorbance at a wavelength of 340 nm. After shaking each plate for 5 s to remove bubbles, absorbance was measured every 15 s at 37°C for 1000 iterations (250 min) or until >50% clot lysis was achieved in all samples.

**Figure 2:**
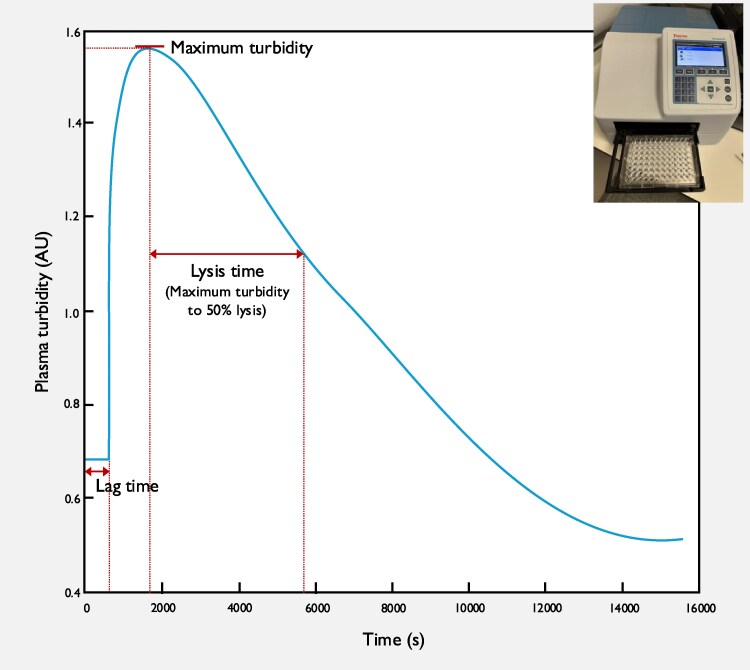
An example of a fibrin clot turbidimetry trace, illustrating the key parameters analysed, generated using 96-well plate reader with capabilities for incubation at 37°C, shaking, and serial absorbance measurement at a wavelength of 340 nm (inset). AU, absorbance units

Automated calculation to determine the time from the beginning of measurement to the start of the increase in turbidity (lag time), the maximum turbidity, and the time from maximum turbidity to 50% clot lysis (lysis time) was performed (*Figure 2*). The mean of duplicate results was used. At all stages during analysis, laboratory staff were blinded to participants’ clinical characteristics, treatment allocation and outcomes.

### Statistical analysis

Statistical analysis was performed by the UCR statistics team. Demographics, characteristics, and biomarker levels at randomization were summarized for the whole substudy cohort and according to quartiles (Q1-4) of lysis time using frequencies for categorical variables and median and 25th and 75th percentiles for continuous variables. The relationships between lysis time and the randomization levels of the biomarkers C-reactive protein (CRP), interleukin (IL)-6, leukocyte count, troponin-T, N-terminal pro-B-type natriuretic peptide (NT-proBNP), growth differentiation factor (GDF)-15, and D-dimer were determined by creating scatter plots and by calculating the Spearman correlation between them.

The number of events and event rates according to the quartile of lysis time were calculated. The outcomes included in this analysis were major or clinically relevant non-major bleeding; and a composite of cardiovascular death, stroke, SE, or MI. Kaplan–Meier curves were constructed and the association between lysis time and outcomes analysed using a Cox proportional hazard model, including adjustment for multiple demographic and clinical variables and biomarker levels (*Table 1*). We based the analyses on the hypothesis that shorter lysis time was associated with increased bleeding risk, but longer lysis time was associated with increased cardiovascular risk. Accordingly, for bleeding outcomes, results were expressed as hazard ratios (HRs) for participants in Q1, Q2, and Q3 compared to Q4. For cardiovascular outcomes, results were expressed as HRs for participants in Q2, Q3 and Q4 compared to Q1. Missing values (5.5%) were imputed using multivariate imputation by chained equations (performed using the *mice* package in R; five imputations were used). Spline functions showing the relationship between outcome and lysis time were created from the Cox models. Restricted cubic splines with 4 knots were used for the unadjusted analyses to balance flexible modelling of potential non-linear relationships with the risk of overfitting for the number of events in the dataset. For the adjusted analyses, three knots were used to accommodate non-linearity while conserving degrees of freedom. Significance was attached to *P*-values of <.05. No adjustment was made for multiple measurements, and so the results should be considered hypothesis-generating. All analyses were performed using R (version 4.4.1).

**Table 1:** Parameters used in multivariate analyses

Demographic	Age, sex, region
Clinical	Weight, systolic blood pressure, history of myocardial infarction, history of spontaneous or clinically relevant bleed, type of atrial fibrillation, history of stroke, transient ischaemic attack or systemic embolism, heart failure, left ventricular ejection fraction <40%, diabetes mellitus, history of hypertension, creatinine
Study participation	Treatment allocation (apixaban vs. warfarin)
Medications	ACE inhibitor or ARB, amiodarone, beta blocker, aspirin, clopidogrel, digoxin, calcium channel blocker, statin, NSAIDs, acid-suppressing drug at randomization
Biomarkers	CRP, IL-6, leukocytes, troponin-T, NT-proBNP, D-dimer

ACE, angiotensin-converting enzyme; ARB, angiotensin receptor blocker; CRP, C-reactive protein; IL-6, interleukin-6; NSAID, non-steroidal anti-inflammatory drug; NT-proBNP, N-terminal pro-B-type natriuretic peptide.

## Results

### Baseline characteristics

The age, sex, clinical characteristics, and comedications of those in the fibrin clot lysis substudy cohort were similar to the main study cohorts, except that exclusion of patients with prior VKA treatment in the substudy cohort was associated with a greater use of aspirin and less use of hydroxymethylglutaryl-CoA reductase inhibitors (*Table 2*). The substudy cohort also had a greater proportion of patients from Europe and lower proportions from North America and Latin America compared to the main study cohorts.

**Table 2:** Baseline characteristics of the fibrin clot lysis substudy relating to demographics, clinical findings, medications and biomarkers

	Fibrin clot lysis substudy cohort *N* = 1841	Main study cohort *N* = 18 201
Demographics		
Age	69 (62–75)	70 (63–76)
Sex:		
Male	1149 (62.4%)	11 785 (64.7%)
Female	692 (37.6%)	6416 (32.3%)
Region:		
Asia/Pacific	274 (14.9%)	2916 (16.0%)
Europe	1119 (60.8%)	7343 (40.3%)
Latin America	173 (9.4%)	3468 (19.1%)
North America	275 (14.9%)	4474 (24.6%)
Treatment allocation		
Apixaban	960 (52.1%)	9120 (50.1%)
Warfarin	881 (47.9%)	9081 (49.9%)
Clinical features		
Weight (kg)	81 (70–94)	82 (70–96)
Systolic blood pressure (mmHg)	130 (120–140)	130 (120–140)
History of myocardial infarction	231 (12.5%)	2585 (14.2%)
Type of atrial fibrillation: paroxysmal	346 (18.8%)	2786 (15.3%)
History of stroke/TIA or SE	307 (16.7%)	3538 (19.4%)
Heart failure	720 (39.1%)	6451 (35.4%)
Diabetes mellitus	424 (23.0%)	4547 (25.0%)
Hypertension	1621 (88.1%)	15 916 (87.4%)
Medications at randomization		
ACE inhibitor or ARB	1302 (70.7%)	12 832 (70.5%)
Amiodarone	231 (12.5%)	2051 (11.3%)
Beta blocker	1150 (62.5%)	11 482 (63.1%)
Aspirin	800 (43.5%)	5632 (30.9%)
Clopidogrel	39 (2.1%)	338 (1.9%)
Digoxin	569 (30.9%)	5828 (32.0%)
Calcium channel blocker	549 (29.8%)	5567 (30.6%)
HMG-CoA reductase inhibitors	650 (35.3%)	8199 (45.0%)
NSAIDs	115 (6.2%)	1520 (8.4%)
Prior use of VKA for >30 days	0 (0%)	10 401 (57.1%)

ACE, angiotensin-converting enzyme; ARB, angiotensin receptor blocker; BMI, body mass index; eGFR, estimated glomerular filtration rate; HMG, hydroxymethylglutaryl; LVEF, left ventricular ejection fraction; NSAIDs, non-steroidal anti-inflammatory drugs; SE, systemic embolism; TIA, transient ischaemic attack.

### Association between lysis time and participant characteristics

Participants with lysis time in the shortest quartile (Q1) were more likely than those with lysis time in the longest quartile (Q4) to be older and male, to have lower weight and BMI, and to have lower estimated glomerular filtration rate (eGFR). They were less likely to have diabetes, hypertension, a history of vascular disease and paroxysmal rather than permanent AF (*Table 3*). Aspirin use was similar across the lysis time quartiles.

**Table 3:** Demographics, clinical features and biomarker levels in each quartile of lysis time

	N	Q1	Q2	Q3	Q4	*P* value
Lysis time (s)		142.5–<1972.5 *N* = 466	1972.5–<2422.5 *N* = 461	2422.5–<2940.0 *N* = 455	2940.0–11302.5 *N* = 459	
Demographics						
Age (years)	1841	70.5 (63.0–77.0)	69.0 (62.0–74.0)	70.0 (62.5–76.0)	66.0 (60.0–72.0)	< .001
Sex: Male	1149	319 (68.5%)	326 (70.7%)	263 (57.8%)	241 (52.5%)	< .001
Female	692	147 (31.5%)	135 (29.3%)	192 (42.2%)	218 (47.5%)	
Treatment allocation						
Apixaban	960	228 (48.9%)	234 (50.8%)	242 (53.2%)	256 (55.8%)	.18
Warfarin	881	238 (51.1%)	227 (49.2%)	213 (46.8%)	203 (44.2%)	
Clinical features						
Weight (kg)	1835	74.0 (62.2–87.5)	80.4 (69.7–92.5)	81.4 (71.0–92.0)	88.2 (77.5–102.4)	< .001
BMI (kg/m^2^)	1832	26.6 (23.4–30.0)	27.8 (24.7–31.2)	28.7 (25.8–32.6)	31.1 (27.7–35.2)	< .001
History of MI	1841	52 (11.2%)	47 (10.2%)	63 (13.8%)	69 (15.0%)	.093
Previous PCI	1841	31 (6.7%)	38 (8.2%)	38 (8.4%)	32 (7.0%)	.68
History of MI, PCI, CABG, or PAD	1841	108 (23.2%)	103 (22.3%)	124 (27.3%)	135 (29.4%)	.043
History of spontaneous or clinically relevant bleeding	1841	56 (12.0%)	52 (11.3%)	56 (12.3%)	54 (11.8%)	.97
Type of AF: paroxysmal	1841	54 (11.6%)	76 (16.5%)	104 (22.9%)	112 (24.4%)	< .001
History of stroke/TIA or SE	1841	71 (15.2%)	83 (18.0%)	76 (16.7%)	77 (16.8%)	.73
Heart failure	1841	179 (38.4%)	171 (37.1%)	169 (37.1%)	201 (43.8%)	.12
LVEF ≤ 40%	1841	88 (18.9%)	73 (15.8%)	51 (11.2%)	60 (13.1%)	.007
Diabetes mellitus	1841	95 (20.4%)	85 (18.4%)	110 (24.2%)	134 (29.2%)	< .001
Creatinine (nmol/L)	1828	1.0 (0.8–1.2)	1.0 (0.9–1.2)	1.0 (0.9–1.2)	1.0 (0.9–1.2)	.050
eGFR (mL/min/1.73 m^2^)	1834	68.9 (53.8–89.2)	76.1 (59.8–92.7)	73.3 (55.1–91.5)	83.6 (66.6–107.7)	< .001
Medications at randomization						
Aspirin	1841	210 (45.0%)	188 (40.8%)	198 (43.4%)	204 (44.4%)	.58
Clopidogrel	1841	13 (2.8%)	11 (2.4%)	7 (1.5%)	8 (1.7%)	.56
ACEi/ARB	1841	311 (66.7%)	317 (68.8%)	316 (69.5%)	358 (78.0%)	<.001
Amiodarone	1841	43 (9.2%)	54 (11.7%)	69 (15.2%)	65 (14.2%)	.028
Beta blocker	1841	277 (59.4%)	269 (58.4%)	288 (63.3%)	316 (68.8%)	.004
Digoxin	1841	143 (30.7%)	131 (28.4%)	130 (28.6%)	165 (35.9%)	.049
Calcium channel blocker	1841	148 (31.8%)	142 (30.8%)	125 (27.5%)	134 (29.2%)	.51
HMG-CoA reductase inhibitors	1841	158 (33.9%)	162 (35.1%)	169 (37.1%)	161 (35.1%)	.78
NSAID	1841	29 (6.2%)	30 (6.5%)	36 (7.9%)	20 (4.4%)	.16
Biomarkers						
CRP (mg/L)	1823	1.7 (0.8–3.9)	1.8 (0.9–3.9)	2.2 (1.0–4.7)	3.1 (1.4–6.2)	< .001
IL-6 (ng/L)	1835	1.9 (1.3–3.4)	1.8 (1.2–3.0)	1.9 (1.3–3.2)	2.1 (1.4–3.6)	.028
Leukocyte count (×10^9^/L)	1785	6.6 (5.5–7.9)	7.0 (5.8–8.1)	6.8 (5.8–8.3)	6.9 (5.8–8.3)	.063
Troponin-T (ng/L)	1822	11.2 (7.6–16.8)	11.1 (7.4–16.2)	11.0 (7.4–15.9)	10.3 (7.7–15.5)	.60
NT-proBNP (ng/L)	1824	828.0 (446.5–1485.0)	713.5 (350.2–1276.8)	650.5 (293.8–1304.2)	590.0 (250.5–1065.0)	< .001
GDF15 (ng/L)	1807	1435.0 (1015.8–2218.0)	1268.0 (965.0–2006.0)	1308.0 (950.0–1965.0)	1318.0 (945.0–1966.0)	.026
D-dimer (µg/L)	1835	621.0 (394.8–1093.2)	550.0 (366.0–892.0)	587.0 (391.8–973.5)	549.0 (345.5–883.5)	.005

Data are median (IQR) or number (percentage).

ACEi/ARB, angiotensin-converting enzyme inhibitor/angiotensin receptor blocker; AF, atrial fibrillation; BMI, body mass index; CABG, coronary artery bypass graft; CRP, C-reactive protein; eGFR, estimated glomerular filtration rate; GDF15, growth differentiation factor 15; HMG, hydroxymethylglutaryl; IL-6, interleukin 6; LVEF, left ventricular ejection fraction; MI, myocardial infarction; NSAID, non-steroidal anti-inflammatory drug; NT-proBNP, N-terminal pro-B-type natriuretic peptide; PAD, peripheral arterial disease; SE, systemic embolism; TIA, transient ischaemic attack.

### Association between lysis time and other biomarkers

Those with lysis time in Q1 had significantly lower CRP and higher NT-proBNP, GDF15, and D-dimer (*Table 3*). However, the correlations between lysis time and other biomarkers were poor (see Supplementary data online, *Figure S1*).

### Association between pre-treatment fibrin clot lysis time and on-treatment clinical outcomes

**Figure 3:**
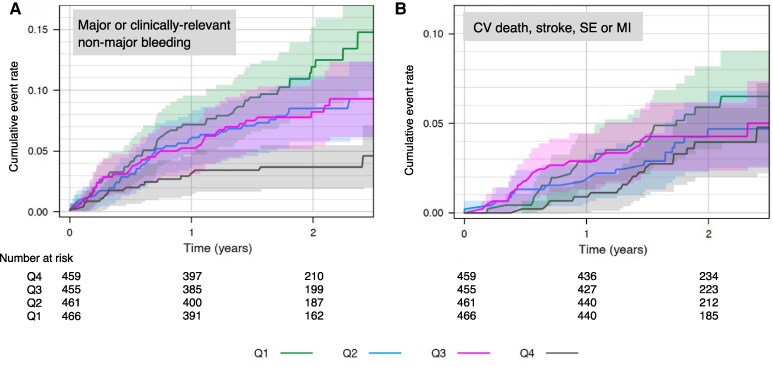
Kaplan–Meier estimate of the cumulative event rates of (*A*) major or clinically relevant non-major bleeding and (*B*) cardiovascular (CV) death, stroke, systemic embolism (SE) or myocardial infarction (MI) by fibrin clot lysis time quartile groups (Q1–4)

**Figure 4:**
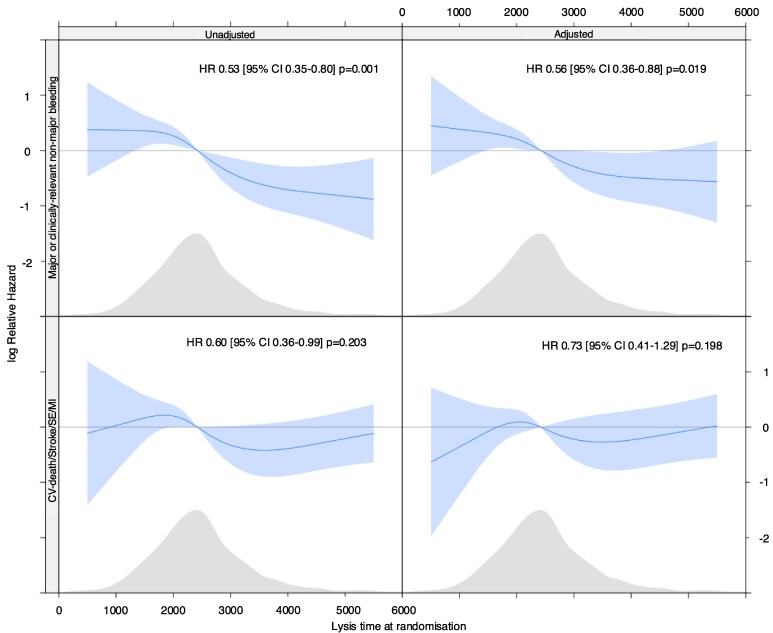
Spline plots derived from Cox regression models (unadjusted and adjusted) demonstrating relationships between lysis time and clinical outcomes. CI, confidence interval; CV, cardiovascular; HR, hazard ratio; MI, myocardial infarction; SE, systemic embolism. Grey shading indicates the distribution of lysis time values

Those in lysis time Q1 had a significantly higher incidence of on-treatment major or clinically relevant non-major bleeding compared to those in Q4, including after adjustment for multiple variables (*Table 4*, *Figures 3* and *4*). There was evidence of a graduated relationship between lysis time and bleeding, with those in Q2 and Q3 having an intermediate risk compared to Q1 and Q4 (*Table 4*). Treatment allocation had no impact on the relationship between lysis time and bleeding risk, whether unadjusted (interaction-*P* = .798) or adjusted (interaction-*P* = .803) (see Supplementary data online, *Figure S2*). Similarly, the relationship was consistent within subgroups of age, sex, BMI and eGFR (see Supplementary data online, *Figure S3*). There was no evidence of significant relationships between lysis time and the incidence of the composite of cardiovascular death, MI, stroke, or SE but event rates were low and confidence intervals were wide (*Table 4*).

**Table 4:** Relationship between lysis time quartiles and clinical outcomes

			Major or clinically relevant non-major bleeding					
					Unadjusted		Adjusted	
Lysis time (s)		*n*	*n*	Rate (%/yr)	HR vs. Q4	*P*	HR vs. Q4	*P*
Q1	142.5–<1972.5	466	51	6.31	2.99 (1.75–5.12)	.001	2.61 (1.45–4.69)	.016
Q2	1972.5–<2422.5	461	39	4.60	2.21 (1.27–3.87)		1.91 (1.06–3.44)	
Q3	2422.5–<2940	455	36	4.31	2.08 (1.18–3.66)		1.85 (1.03–3.32)	
Q4	2940–11302.5	459	18	2.06	-		-	

			Cardiovascular death, stroke, systemic embolism or MI					
					Unadjusted		Adjusted	
Lysis time (s)		N	n	Rate (%/yr)	HR vs. Q1	*P*	HR vs. Q1	*P*
Q1	142.5–<1972.5	466	27	3.02	-	.492	-	.987
Q2	1972.5–<2422.5	461	19	2.08	0.69 (0.38–1.23)		0.97 (0.52–1.82)	
Q3	2422.5–<2940	455	21	2.30	0.96 (0.43–1.34)		1.03 (0.55–1.95)	
Q4	2940–11302.5	459	19	2.05	0.67 (0.37–1.21)		1.10 (0.55–2.21)	

Hazard ratios (HR) with 95% confidence intervals calculated using Q4 (for bleeding) or Q1 (for cardiovascular events) as reference. MI, myocardial infarction.

### Associations between pre-treatment fibrin clot lag time or maximum turbidity and on-treatment clinical outcomes

There were no significant associations between either lag time or maximum turbidity and either rates of major or clinically relevant non-major bleeding or cardiovascular death, MI, stroke or SE (see Supplementary data online, *Tables S3* and *S4*), despite the associations between maximum turbidity and inflammatory markers (see Supplementary data online, *Figures S4* and *S5*).

## Discussion

The association of fibrin clot dynamics with clinical outcomes in patients with cardiovascular disease has gained growing attention, with fibrinolysis potential increasingly recognized as an important biomarker of cardiovascular risk. It has previously been demonstrated that a longer lysis time is significantly associated with worse cardiovascular outcomes in patients receiving dual antiplatelet therapy (DAPT) for ACS, including in patients with diabetes mellitus who tend to have impairment of fibrinolysis.^10,11,16^ Here, we have shown that a shorter pre-treatment lysis time is significantly associated with a higher risk of major or clinically relevant non-major bleeding in patients receiving OAC for AF. This effect was similar whether participants received apixaban or warfarin. Lysis time in the highest quartile was associated with a significantly lower risk of major or clinically relevant non-major bleeding, even after adjustment for multiple clinical variables and biomarkers known to be of prognostic importance or relevant to drug pharmacokinetics. Furthermore, there was evidence of a progressive reduction in bleeding risk with increasing lysis time, with bleeding events more common in those with lysis time in Q1 than in Q2 and Q3 and less common in Q4 (*Structured Graphical Abstract*).

Assessment of bleeding risk is a key aspect of decision-making around the initiation of OAC for AF. Current scores include either clinical features alone or a combination of clinical features and biomarkers.^6,17^ Our data suggest that pre-treatment lysis time may be another candidate biomarker for inclusion in models of bleeding risk prediction, though future work is needed to explore this further. Our findings are supported by the fact that tranexamic acid, which inhibits fibrinolysis, is known to improve haemostasis in a range of settings, including in patients receiving OAC.^18^

In this study, there was no strong evidence of a relationship between lysis time and cardiovascular events, with no finding of an association that remained statistically significant in both the unadjusted and adjusted analyses. Conversely, in the setting of ACS, there is now a good body of evidence that hypofibrinolysis is associated with worse ischaemic outcomes.^10,11,19^ While this may suggest that dynamics of fibrinolysis are less important predictors of cardiovascular outcomes in patients with AF receiving OAC than in patients with ACS receiving DAPT, it is important to note that the event rates for the cardiovascular outcomes were low in this substudy cohort, in part because a reduced number of samples from patients with stroke were available, and so further work with greater statistical power is required to explore this area. Furthermore, within lysis time Q4 (2940 to 11 302 s), there was a numerical trend towards higher rates of cardiovascular events with increasing lysis time on adjusted analysis (*Figure 3*) that was consistent with previous findings in ACS patients and warrants future scrutiny of those with very high lysis times. It remains possible, however, that differences in the pathophysiology of thrombosis between arterial and atrial sites may also explain the discrepant findings between the AF and ACS populations. Arterial thrombus is more likely to be ‘white’ in nature, with a high fibrin and platelet content.^20^ Conversely, thrombus formed in sites of lower shear stress, including the atria, is more likely to be ‘red’ with a preponderance of erythrocytes and relatively less dense fibrin formation.^21^ It may therefore be that parameters of fibrin turnover are more determinant of cardiovascular outcomes in those at greater risk of arterial rather than cardioembolic events. Alternatively, or in addition, it may be that anticoagulation but not antiplatelet therapy reduces the impact of a longer pre-treatment lysis time on cardiovascular risk.

We also compared participant characteristics and biomarkers between lysis time quartiles. Inflammatory markers were lower in those with shorter lysis time, consistent with prior literature.^22^ Levels of GDF15 were higher in those with shorter lysis time, consistent with the fact that GDF15 is known to positively correlate with bleeding risk, including in the ARISTOTLE trial.^23^ Despite this association between GDF15 and lysis time, the overall correlation between GDF15 level and lysis time was poor, and lysis time remained of statistical significance as a predictor of bleeding even after including GDF15 in a *post hoc* multivariable adjustment along with other clinical characteristics and biomarkers associated with bleeding risk (see Supplementary data online, *Table S2*).

Paroxysmal rather than persistent or permanent AF was significantly less frequent in those with a lysis time in Q1, around half as common as those in Q4. Though confounders cannot be excluded, this is difficult to explain. Further exploration is needed given paroxysmal AF is associated with lower levels of inflammation than other types of AF,^24^ though sampling may not capture transient inflammatory stimuli associated with an arrhythmic episode in a patient with paroxysmal AF. Whilst greater BMI, IL-6, CRP and presence of diabetes were associated with longer lysis time in our analysis, previous data from the ARISTOTLE trial suggest these factors do not appear to influence whether AF is paroxysmal or permanent.^25^

Though we focussed on pre-treatment fibrin clot dynamics in this analysis, future studies of on-treatment fibrin clot turbidimetry may provide further insights into bleeding and ischaemic risk in this population. Though not fully explored, there is some evidence that therapeutic doses of oral anticoagulation reduce lysis time, probably mediated by reducing activation of thrombin-activatable fibrinolysis inhibitor,^26,27^ and that on-treatment lysis time may be associated with clinical outcomes in patients with AF.^28^

Similarly, more work to understand the mechanisms behind variation in lysis time, including genetic factors, is warranted. Other medications taken by patients with cardiovascular disease such as HMG-CoA reductase inhibitors or aspirin might influence clot composition and lysis,^29,30^ but, in our study, their use was similar between the lysis time quartiles and was adjusted for in the multivariable model.

### Limitations

The sample size of the substudy cohort means interpretation of the results should be tempered against any potential for limited statistical power. As a subset of plasma samples from participants with stroke events were not available for analysis, this contributed to the reduced power of this analysis to detect significant associations between lysis time and stroke alone or the composite of cardiovascular events. Our substudy cohort also comprised a higher proportion of European patients and fewer Latin American and North American participants compared to the main trial population, which may potentially introduce geographical bias. Plasma samples were stored at −80°C for around 10–12 years prior to analysis, but unpublished data show samples for fibrin clot turbidimetry remain stable at this point, and furthermore, normal pooled plasma was used for performing each assay to ensure the patient sample responses were broadly consistent with those from more recently stored plasma.

## Conclusions

Shorter pre-treatment lysis time was associated with significantly increased risk of major or clinically relevant non-major bleeding in patients receiving OAC for AF. Measurement of pre-treatment lysis time may aid in the determination of on-treatment bleeding risk in this population.

## Supplementary Material

ehaf347_Supplementary_Data

## Supplementary data

### Pre-registered Clinical Trial Number

The pre-registered clinical trial number for the ARISTOTLE study and its biomarker substudy is EudraCT number 2006-002147-91.

## Declarations

### Data Availability

Original data are provided in this manuscript. The authors will consider proposals for further analyses of the extensive ARISTOTLE main study and biomarker substudy database.
