# Supplementary material for: Pre-treatment lysis time of plasma-derived fibrin clots and bleeding in patients on oral anticoagulants for atrial fibrillation in the ARISTOTLE trial
Source: Eur Heart J. 2025 May 30;47(16):1949–60. doi: 10.1093/eurheartj/ehaf347 (PMC13099228; doi:10.1093/eurheartj/ehaf347)
Supplement: ehaf347_Supplementary_Data [file ehaf347_supplementary_data.docx]

**SUPPLEMENTARY APPENDIX**

**
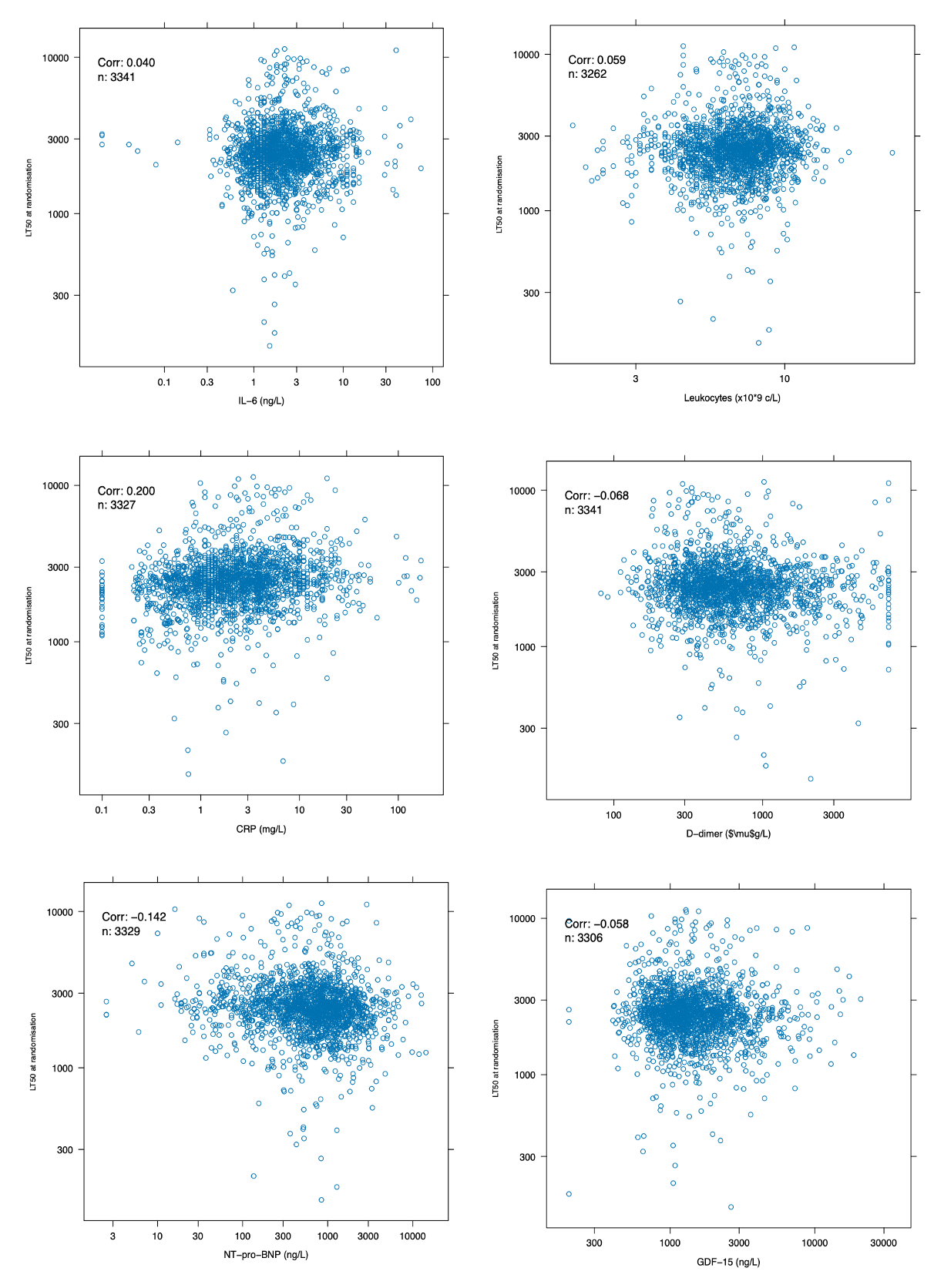
**

**Supplementary Figure 1.** **Correlations between lysis time (LT50) and other biomarkers.**

Corr, Spearman correlation R value; CRP, C-reactive protein; GDF-15, growth differentiation factor 15; IL-6, interleukin 6; NT-pro-BNP, N-terminal-pro-B-type natriuretic peptide.

**
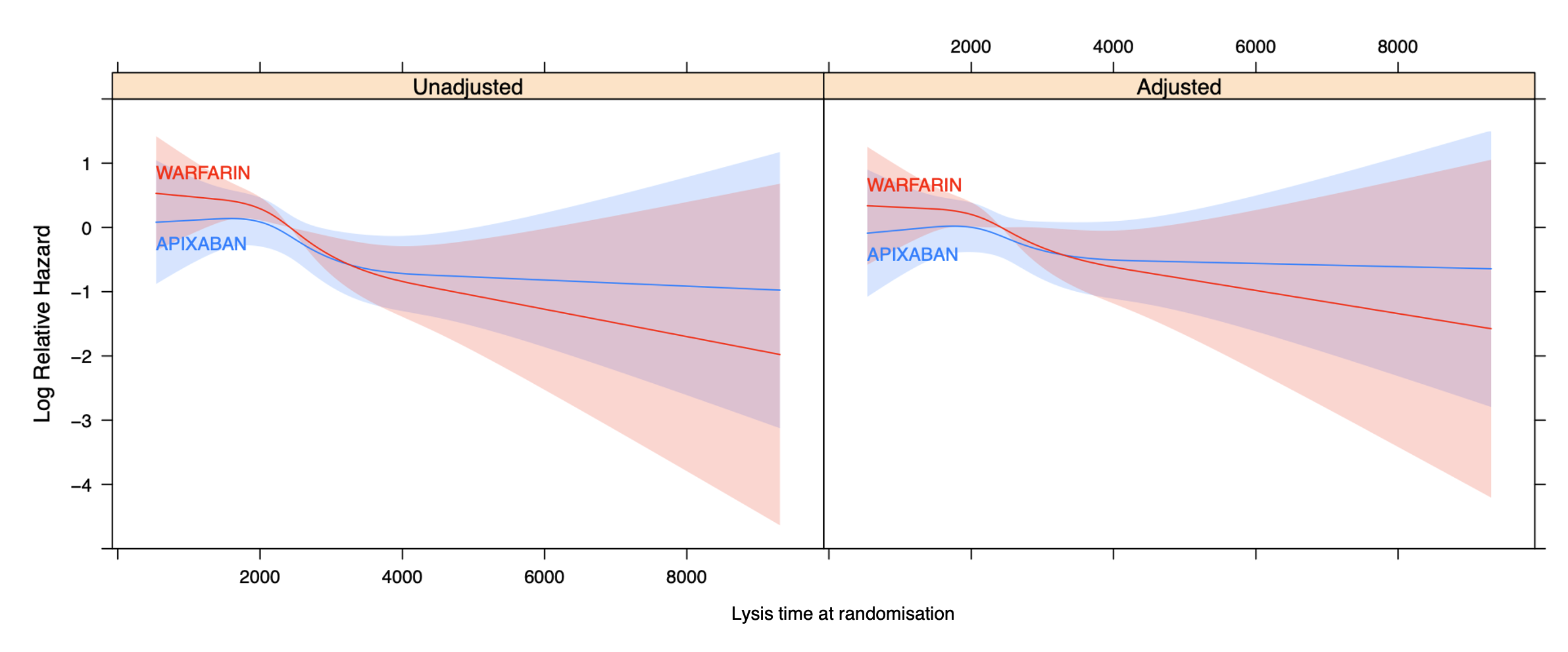
**

**Supplementary Figure 2.** **Relationship between lysis time at randomisation and risk of major or clinically-relevant non-major bleeding in those allocated to receive apixaban or warfarin.**


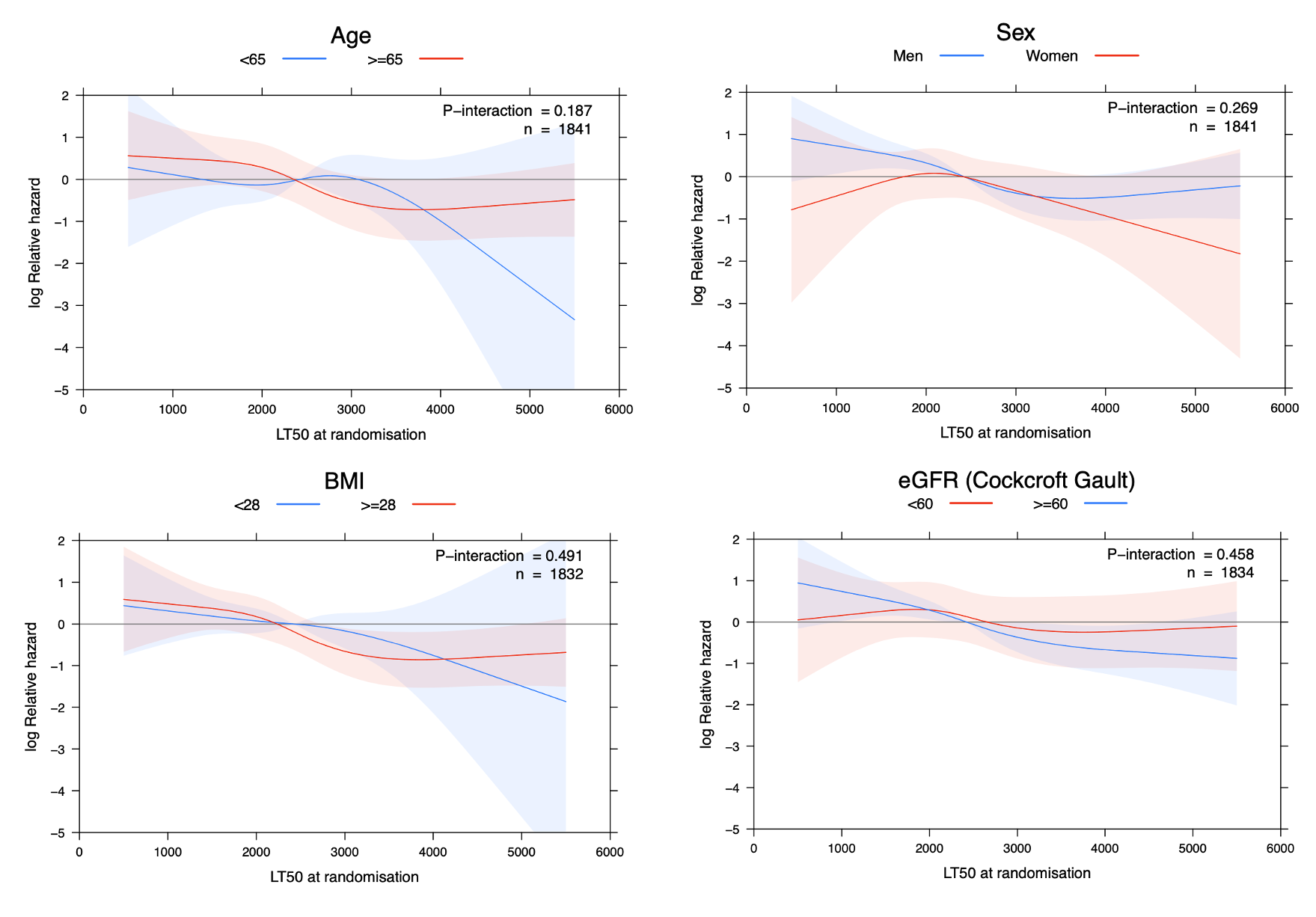


**Supplementary Figure 3. Relationship between lysis time (LT50) at randomisation and relative hazard of major or clinically-relevant non-major bleeding, stratified by age (<65 and ≥65 years), sex, body mass index (BMI, <28 and ≥28 kg/m^2^) and estimated glomerular filtration rate (eGFR, <60 and ≥60 ml/min).**

**
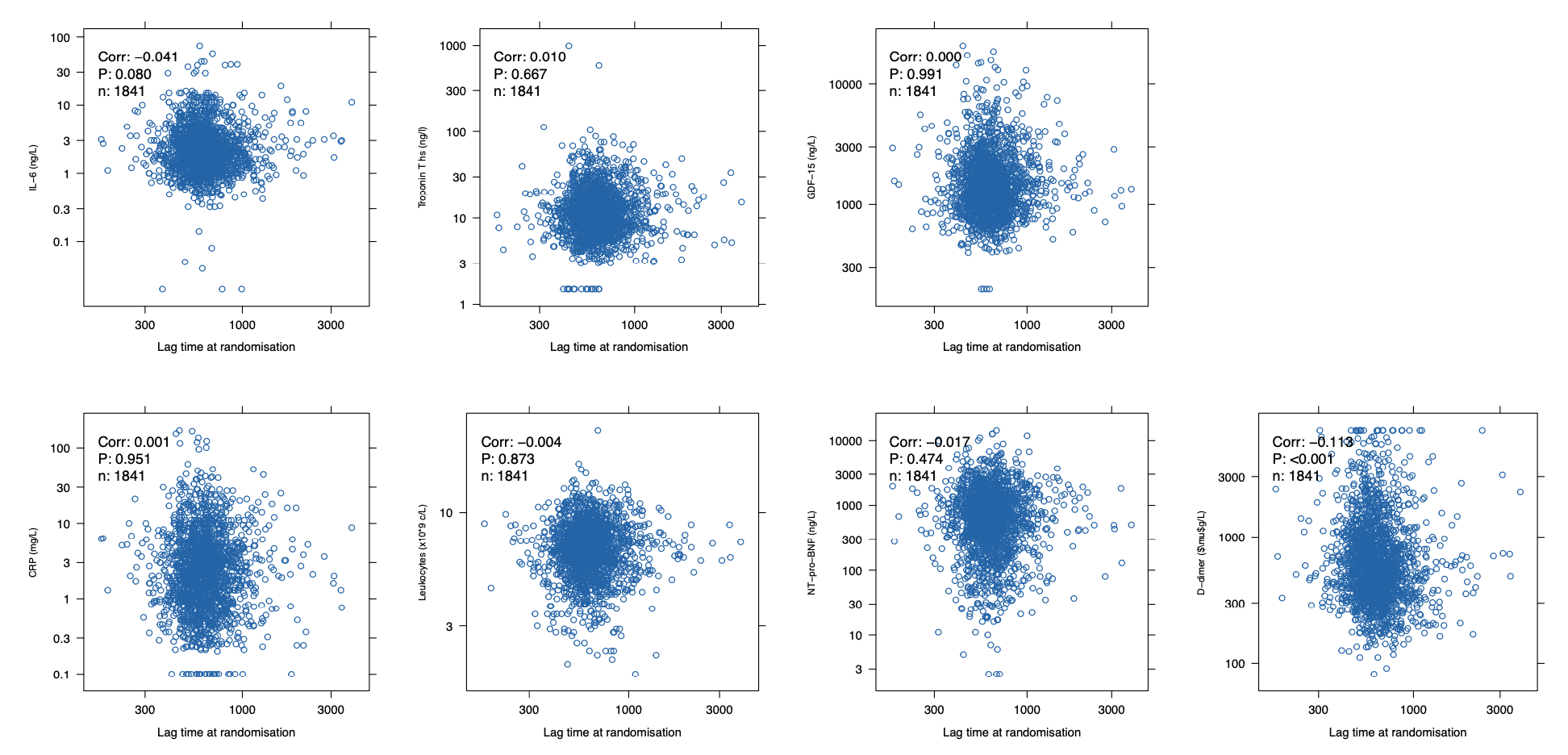
**

**Supplementary Figure 4. Scatterplots showing the relationship between lag time and biomarkers.** Spearman correlations are included.

**
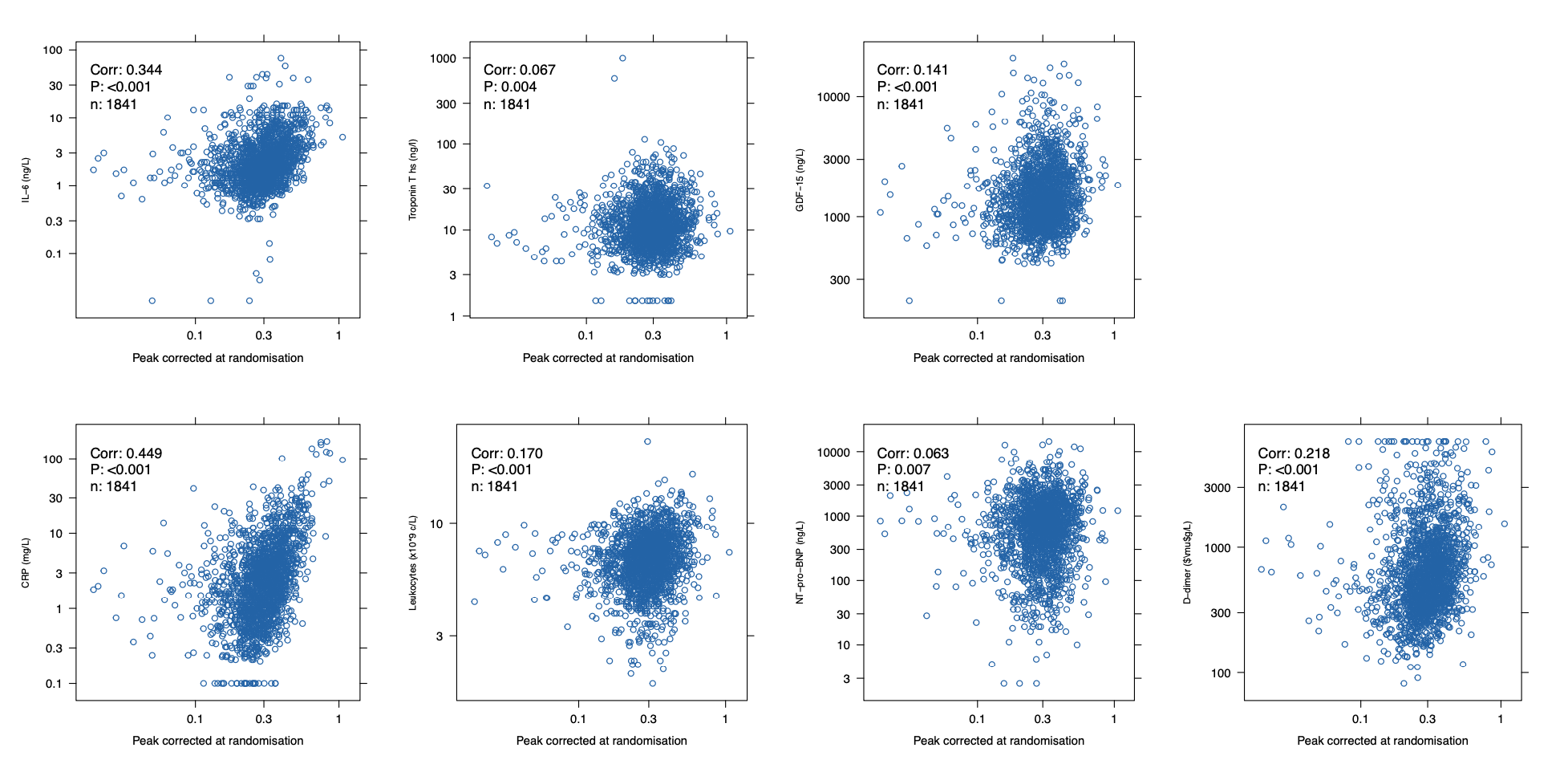
**

**Supplementary Figure 5. Scatterplots showing the relationship between maximum turbidity and biomarkers.** Spearman correlations are included.

**Supplementary Table 1. Number of events and event rate in %/year (per 100 person-years), split by quartiles of pre-treatment lysis time.**

CV, cardiovascular; SE, systemic embolism

*CV-death, stroke/SE, myocardial infarction.

**Major and non-major clinically-relevant bleeding

| **Lysis time (s)** | 142.5 – <1972.5 | 1972.5 – <2422.5 | 2422.5 – <2940 | 2940 – 11302.5 |
| --- | --- | --- | --- | --- |
| **n** | 466 | 461 | 455 | 459 |
| **Composite CV events* (n)** | 27 | 19 | 21 | 19 |
| **Composite CV event rate (%/year)** | 3.02 | 2.08 | 2.30 | 2.05 |
| **Stroke/SE (n)** | 6 | 5 | 1 | 2 |
| **Stroke/SE rate (%/year)** | 0.67 | 0.55 | 0.11 | 0.21 |
| **CV-death (n)** | 23 | 17 | 17 | 14 |
| **CV-death rate (%/year)** | 2.53 | 1.83 | 1.84 | 1.49 |
| **Bleeding events** (n)** | 51 | 39 | 36 | 18 |
| **Bleeding event rate (%/year)** | 6.31 | 4.60 | 4.31 | 2.06 |
| **All-cause death (n)** | 44 | 25 | 29 | 31 |
| **All-cause death (%/year)** | 4.85 | 2.70 | 3.14 | 2.06 |

**Supplementary Table 2.** **Unadjusted and adjusted rates of major or clinically-relevant non-major bleeding by lysis time quartile**

|  | | | **Major or clinically-relevant non-major bleeding** | | | | | | | |
| --- | --- | --- | --- | --- | --- | --- | --- | --- | --- | --- |
|  |  |  |  | | **Unadjusted** | | **Adjusted** | | **Adjusted including GDF-15** | |
| **Lysis time (s)** | | **n** | **n** | **Rate (%/yr)** | **HR vs Q4** | **P** | **HR vs Q4** | **P** | **HR vs Q4** | **P** |
| Q1 | 142.5 - <1972.5 | 466 | 51 | 6.31 | 2.99  (1.74-5.11) | 0.001 | 2.61  (1.45-4.69) | 0.016 | 2.59  (1.45-4.69) | 0.018 |
| Q2 | 1972.5 - <2422.5 | 461 | 39 | 4.60 | 2.21  (1.27-3.87) |  | 1.91  (1.06-3.44) |  | 1.92  (1.06-3.48) |  |
| Q3 | 2422.5 - <2940 | 455 | 36 | 4.31 | 2.08  (1.18-3.66) |  | 1.85  (1.03-3.32) |  | 1.89  (1.05-3.40) |  |
| Q4 | 2940-11302.5 | 459 | 18 | 2.06 | - |  | - |  | - |  |

**Supplementary Table 3.** **Unadjusted and adjusted rates of (A) major or clinically-relevant non-major bleeding and (B) cardiovascular death, stroke, systemic embolism or myocardial infarction by lag time quartile.**

|  | | | **Major or clinically-relevant non-major bleeding** | | | | | |
| --- | --- | --- | --- | --- | --- | --- | --- | --- |
|  |  |  |  | | **Unadjusted** | | **Adjusted** | |
| **Lag time (s)** | | **n** | **n** | **Rate (%/yr)** | **HR vs Q4** | **P** | **HR vs Q4** | **P** |
| Q1 | 174.5 – <529 | 462 | 38 | 4.51 | 0.93 (0.60–1.45) | 0.698 | 0.68 (0.42–1.09) | 0.146 |
| Q2 | 529 – <614.5 | 462 | 32 | 3.77 | 0.78 (0.49–1.24) |  | 0.58 (0.36–0.94) |  |
| Q3 | 614.5 – <729.5 | 459 | 33 | 4.00 | 0.82 (0.52–1.30) |  | 0.68 (0.43–1.10) |  |
| Q4 | 729.5–3874.5 | 458 | 41 | 4.85 | - |  | - |  |
|  | | | **Cardiovascular death, stroke, systemic embolism or MI** | | | | | |
|  |  |  |  | | **Unadjusted** | | **Adjusted** | |
| **Lag time (s)** | | **N** | **n** | **Rate (%/yr)** | **HR vs Q1** | **P** | **HR vs Q1** | **P** |
| Q1 | 174.5 – <529 | 462 | 26 | 2.90 | - | 0.053 | - | 0.063 |
| Q2 | 529 – <614.5 | 462 | 18 | 1.94 | 0.66 (0.36–1.21) |  | 0.66 (0.36–1.22) |  |
| Q3 | 614.5 – <729.5 | 459 | 29 | 3.21 | 1.09 (0.64–1.85) |  | 1.22 (0.70–2.15) |  |
| Q4 | 729.5 – <3874.5 | 458 | 13 | 1.42 | 0.48 (0.25–0.94) |  | 0.55 (0.27–1.13) |  |

**Supplementary Table 4.** **Unadjusted and adjusted rates of (A) major or clinically-relevant non-major bleeding and (B) cardiovascular death, stroke, systemic embolism or myocardial infarction by maximum turbidity quartile.**

|  | | | **Major or clinically-relevant non-major bleeding** | | | | | |
| --- | --- | --- | --- | --- | --- | --- | --- | --- |
|  |  |  |  | | **Unadjusted** | | **Adjusted** | |
| Maximum turbidity (AU) | | **n** | **n** | **Rate (%/yr)** | **HR vs Q4** | **P** | **HR vs Q4** | **P** |
| Q1 | 0.0195 – <0.24 | 461 | 31 | 3.63 | 0.69 (0.43–1.09) | 0.398 | 0.79 (0.47–1.35) | 0.849 |
| Q2 | 0.24 – <0.301 | 460 | 34 | 4.04 | 0.76 (0.49–1.19) |  | 0.85 (0.51–1.42) |  |
| Q3 | 0.301 – <0.37 | 460 | 35 | 4.18 | 0.79 (0.51–1.23) |  | 0.87 (0.54–1.39) |  |
| Q4 | 0.37 – 1.06 | 460 | 44 | 5.30 | - |  | - |  |
|  | | | **Cardiovascular death, stroke, systemic embolism or MI** | | | | | |
|  |  |  |  | | **Unadjusted** | | **Adjusted** | |
| Maximum turbidity (AU) | | **N** | **n** | **Rate (%/yr)** | **HR vs Q1** | **P** | **HR vs Q1** | **P** |
| Q1 | 0.0195 – <0.24 | 461 | 21 | 2.32 | - | 0.887 | - | 0.801 |
| Q2 | 0.24 – <0.301 | 460 | 19 | 2.07 | 0.90 (0.48–1.67) |  | 0.93 (0.49–1.78) |  |
| Q3 | 0.301 – <0.37 | 460 | 24 | 2.62 | 1.14 (0.64–2.06) |  | 1.04 (0.55–1.96) |  |
| Q4 | 0.37 – 1.06 | 460 | 22 | 2.42 | 1.05 (0.58–1.91) |  | 0.76 (0.38–1.53) |  |
